# Cardiovascular risk factors and memory decline in middle-aged and older adults: the English Longitudinal Study of Ageing

**DOI:** 10.1186/s12877-019-1350-5

**Published:** 2019-12-02

**Authors:** Beatriz Olaya, Maria Victoria Moneta, Martin Bobak, Josep Maria Haro, Panayotes Demakakos

**Affiliations:** 1 Research, Innovation and Teaching Unit, Parc Sanitari Sant Joan de Déu, Fundació Sant Joan de Déu, Universitat de Barcelona, Sant Boi de Llobregat, Spain; 2 Instituto de Salud Carlos III, Centro de Investigación Biomédica en Red de Salud Mental (CIBERSAM), Madrid, Spain; 3 Department of Epidemiology and Public Health, University College London, London, UK

**Keywords:** cardiovascular risk factors, population-based survey, age-dependent effect, Memory decline

## Abstract

**Background:**

We investigated the association between trajectories of verbal episodic memory and burden of cardiovascular risk factors in middle-aged and older community-dwellers.

**Methods:**

We analysed data from 4372 participants aged 50–64 and 3005 persons aged 65–79 years old from the English Longitudinal Study of Ageing who were repeatedly evaluated every 2 years and had six interviews of a 10-year follow-up. We measured the following baseline risk factors: diabetes, hypertension, smoking, physical inactivity and obesity to derive a cardiovascular risk factor score (CVRFs). Adjusted linear mixed effect regression models were estimated to determine the association between number of CVFRs and six repeated measurements of verbal memory scores, separately for middle-aged and older adults.

**Results:**

CVRFs was not significantly associated with memory at baseline. CVFRs was significantly associated with memory decline in middle-aged (50-64y), but not in older (65-79y) participants. This association followed a dose-response pattern with increasing number of CVFRs being associated with greater cognitive decline. Comparisons between *none* versus some CVRFs yielded significant differences (*p* < 0.05).

**Conclusions:**

Our findings confirm that the effect of cumulative CVRFs on subsequent cognitive deterioration is age-dependent. CVRFs are associated with cognitive decline in people aged 50–64 years, but not in those aged ≥65 years. Although modest, the memory decline associated with accumulation of cardiovascular risk factors in midlife may increase the risk of late-life dementia.

## Background

Cardiovascular risk factors (CVRFs) (tobacco smoking, hypertension, obesity, physical inactivity and diabetes) are highly prevalent among midlife and older adults [1], and constitute leading causes of mortality. Of the 2.5 million Americans who died in 2005, tobacco smoking killed 1 in 5, elevated blood pressure was responsible for 1 in 6 deaths, and obesity and physical inactivity took 1 in 10 lives [2]. Besides this augmented risk for death, population-based studies have also identified CVRFs as strongly related to higher risk for accelerated cognitive decline [3, 4] and dementia [5]. For example, current smokers are between 50 and 80% more likely to develop Alzheimer’s Disease (AD) in the future, compared with those who never smoked [6, 7], and older adults who engage in high leisure-time physical activity have approximately 45% less risk of AD than people with the lowest level [8]. The effect of CVRFs on cognitive deterioration is mostly, but not exclusively, driven by cardiovascular diseases (CVD) [9]. Other pathophysiological mechanisms through which CVRFs might impact on cognitive decline or risk for dementia include inflammation and oxidative stress, cerebral small vessel diseases, cerebral hypoxia and hypoperfusion, or neurodegeneration in the brain, which in turn might also increase the risk for heart disease [10]. Several epidemiological studies have shown that the effect of several CVRFs on cognitive deterioration follows a dose-response pattern [10]. Thus, composite scores of CVRFs are often used as potential tools to detect at-risk older adults for cognitive decline and dementia.

Despite the association between CVRFs in older adults and risk for dementia being well-established [5, 9, 11], several authors postulate that this relationship might be age-dependent [12]. High blood pressure during midlife has been shown to be associated with an increased risk of AD in late life [13, 14], whereas research on the effect of high blood pressure in late life and dementia has yielded mixed results [9]. Similarly, the association of obesity and AD seems to be also age-dependent, with obesity in midlife reported as risk factor for AD [15], and underweight in late-life being more important in predicting AD [16]. Other studies have also demonstrated that aggregated CVRFs at midlife are associated with cognitive decline in the middle age [17, 18], suggesting that this effect can be observed at early stages. The associations between CVRFs during late life and dementia are less clear, with several studies finding very small or null effects on subsequent cognitive deterioration [13, 19].

Comparison across these studies focusing on middle-age versus late-life might be challenging because of distinct study designs, measures and sampling characteristics. To the authors’ knowledge, no studies have compared the impact of a summary score of CVRFs on subsequent cognitive deterioration in population-based middle aged versus older cohorts. The impact of a summary score of CVRFs at baseline on subsequent trajectories of cognition (verbal episodic memory) over a period of 10 years was investigated in a longitudinal, nationally-representative cohort of adults aged 50 to 64 and 65 to 79 years old. We aimed to study whether this association followed a dose-response pattern by exploring the interaction between time and CVRFs. Models were adjusted for several confounders to determine whether this association was above and beyond the effect of potential explanatory variables. The stratification of models by two age cohorts (middle-aged and older adults) allowed us explore age differences in the association between CVRFs and cognitive decline.

## Methods

### Study population

This study analysed data from the English Longitudinal Study of Ageing (ELSA), an ongoing project intended to collect data to study the dynamics of health, social, wellbeing and economic circumstances in the English population aged 50+, and more details about the study can be found elsewhere [20]. Briefly, it is a longitudinal, nationally representative survey of people aged 50 years and older living in private addresses in England. It was a randomized, stratified, multi-stage sample of participants [20] who earlier had taken part of the Health Survey England (1998, 1999 and 2001). The baseline sample (11,391 core members) was first assessed in 2002–03 and re-assessed every 2 years. Data are freely available from the UK Data Archive (https://beta.ukdataservice.ac.uk).

The present study used data from 6 successive waves of ELSA over 10 years of follow-up. We focused on core members who completed a non-proxy interview at baseline (*n* = 11,233) and excluded those who were aged 80+ at baseline (*n* = 10,026). Eighty-two participants who reported at baseline to have been diagnosed by a doctor with AD, Parkinson’s disease, dementia, organic brain syndrome, senility or any other serious memory impairment were excluded. Participants with information on verbal episodic memory score at baseline and at least in another wave and with complete information in all covariates (age, gender, educational and wealth level, marital status, number of CVD and non-CVD diseases) were included in the analysis, resulting in a final *n* of 7377.

### Measures

#### Outcomes

The ELSA study includes a battery of cognitive tests to measure cognitive functioning in the elderly [21]. We focused on verbal episodic memory, which is related to every-day activities of older adults and has been shown to be sensitive to age-related decline [22]. The measure was assessed in the six waves using both immediate and delayed word recall tasks in which ten common words were presented aurally by computer to ensure standardised delivery. Participants were asked to recall them immediately and after a short delay, which was filled with other cognitive tests. There were four alternative lists, so that different lists could be given in distinct waves. The number of correct responses was recorded each time. This approach has been used elsewhere [21], and the word lists used here were those developed for the Health Retirement Study (HRS) [23]. The number of recalled words from both tests was added to obtain a total memory score (ranging from 0 to 20) with higher scores indicating better memory. The correlation coefficients between the immediate and delayed recall at baseline was 0.65. Immediate and delayed recalls have been known to have good construct validity and consistency [24].

##### Cardiovascular risk factor score

Following the literature [11], we generated a summary score, which included the following baseline CVRFs: self-reported hypertension and diabetes, history of smoking (current or ever vs. never), objectively measured obesity (Body Mass Index (BMI) > 30 kg/m^2^) and physical inactivity (not at all or mild physical activity vs. moderate or vigorous physical activity at least once a week). As BMI was not measured at baseline in 2002–03, we used BMI data that were measured in the Health Survey for England in 1998, 1999 and 2001. The CVRFs score initially ranged from 0 to 5. Due to the small number of people with 4 and 5 CVRFs, these categories were collapsed, and finally the CVRFs score had four categories, ranging from 0 to ≥3 CVRFs.

##### Covariates

Covariates were measured at baseline and included sex, age, marital status (never married, legally separated or divorced, married/remarried, and widowed), education (A-level or above recorded as *high*; O-level/Secondary education recorded as *medium* level; and no qualifications recorded as *low* education), quintiles of total net non-pension household wealth (including the net worth of savings and investments, property and business assets, but not pension-related assets), number of self-reported CVDs (angina, heart attack, congestive heart, abnormal heart rhythm, and stroke) and non-CVDs (chronic lung diseases, asthma, arthritis, osteoporosis, and cancer). Summary scores of CVDs and non-CVDs were generated. Depressive symptoms were assessed using the short 8-item Centre for Epidemiologic Studies Depression Scale (CES-D) [25]. We calculated a summary CES-D score using all eight binary (Yes/No) symptom items. The CES-D summary score ranged from 0 to 8, with higher scores indicating more depressive symptoms.

### Statistical analysis

Unadjusted and adjusted linear mixed effect regression models were estimated separately for the middle-aged (50–64 years) and older cohorts (65–79 years). The adjusted models included the covariates aforementioned. Time as continuous variable (from 0 to 10 years) was included in the models. To account for non-response, including survivor bias [26], we adjusted our models for a variable distinguishing between *completers* (participants who had complete memory data in all visits) and *drop-outs* (participants who missed at least one wave of ELSA for any reason including intermittent attrition, death and drop-out). To explore whether the rate of memory decline was different across the number of CVRFs, the interaction CVRFs*time was included in the adjusted models. Intercepts and slopes were measured as random effects. Since CVRFs are highly correlated with the presence of CVDs, adjustment for CVDs may attenuate the association of CVRFs with cognition. Thus, we also calculated the adjusted models excluding this variable (see Additional file 1: Table S1).

Marginal effects were used to graph linear adjusted predictions of verbal episodic memory over the follow-up while holding covariates constant. Post-hoc corrections (Bonferroni) for multiple comparisons were performed.

Analyses were conducted with SAS 9.4 (SAS Institute, Cary NC) using the PROC MIXED procedure and Stata (SE version 13, College Station, TX). A two-side *p* value of less than 0.05 was considered as statistically significant.

## Results

Table 1 presents a descriptive analysis of the total sample (*N* = 7377) and by age cohorts.

**Table 1:** Descriptive characteristics of the overall sample (*N* = 7377) and by age groups

Age group				
	Overall sample	50–64	65–79	*P value*
	*N* = 7377	*n* = 4372 (59.3%)	*n* = 3005 (40.7%)	
Socio-demographics characteristics				
Female, *n (%)*	3996 (54.2%)	2349 (53.7%)	1647 (54.8%)	.360
Age, *mean (SD)*	62.6 (8.1)	56.8 (4.1)	71 (4.1)	<.001
Marital status, *n (%)*				
Never married	380 (5.2%)	244 (5.6%)	136 (4.5%)	<.001
Married/remarried	5190 (70.4%)	3257 (74.5%)	1933 (64.3%)	
Separated/divorced	870 (11.8%)	632 (14.5%)	238 (7.9%)	
Widowed	937 (12.7%)	239 (5.5%)	698 (23.2%)	
Education level, *n (%)*				<.001
Low	2785 (37.8%)	1304 (29.8%)	1481 (49.3%)	
Medium	2269 (30.8%)	1408 (32.2%)	861 (28.7%)	
High	2323 (31.5%)	1660 (38.0%)	663 (22.1%)	
Quintiles of wealth, *n (%)*				<.001
Lowest	1163 (15.8%)	596 (13.6%)	567 (18.9%)	
2nd	1405 (19.1%)	808 (18.5%)	597 (19.9%)	
3rd	1516 (20.6%)	904 (20.7%)	612 (20.4%)	
4th	1625 (22%)	992 (22.7%)	633 (21.1%)	
Highest	1668 (22.6%)	1072 (24.5%)	596 (19.8%)	
Cardiovascular risk factors				
High blood pressure, *n (%)*	2671 (36.2%)	1340 (30.6%)	1331 (44.3%)	<.001
Diabetes, *n (%)*	476 (6.5%)	205 (4.7%)	271 (9.0%)	<.001
Obesity, *n (%)*	1914 (26%)	1128 (25.8%)	786 (26.2%)	.732
Currently or past smoking, *n (%)*	4711 (63.9%)	2784 (63.7%)	1927 (64.1%)	.694
Physical inactivity, *n (%)*	1427 (19.3%)	714 (16.3%)	713 (23.7%)	<.001
Number of cardiovascular risk factors, *n (%)*				<.001
None	1172 (15.9%)	785 (18.0%)	387 (12.9%)	
One	2876 (39%)	1810 (41.4%)	1066 (35.5%)	
Two	2014 (27.3%)	1130 (25.8%)	884 (29.4%)	
Three or more	1315 (17.8%)	647 (14.8%)	668 (22.2%)	
Non-CVDs				
Chronic lung disease, *n (%)*	440 (6%)	214 (4.9%)	226 (7.5%)	<.001
Asthma, *n (%)*	852 (11.6%)	519 (11.9%)	333 (11.1%)	0.297
Arthritis, *n (%)*	2241 (30.4%)	1129 (25.8%)	1112 (37.0%)	<.001
Osteoporosis, *n (%)*	327 (4.4%)	132 (3.0%)	195 (6.5%)	<.001
Cancer, *n (%)*	413 (5.6%)	204 (4.7%)	209 (7.0%)	<.001
Number of non-CVDs, *n (%)*				<.001
None	4081 (55.3%)	2655 (60.7%)	1426 (47.5%)	
One	2484 (33.7%)	1319 (30.2%)	1165 (38.8%)	
Two or more	812 (11%)	398 (9.1%)	414 (13.8%)	
CVDs				
Angina, *n (%)*	629 (8.5%)	232 (5.3%)	397 (13.2%)	<.001
Heart attack, *n (%)*	379 (5.1%)	138 (3.2%)	241 (8.0%)	<.001
Congestive heart failure, *n (%)*	48 (0.7%)	13 (0.3%)	35 (1.2%)	<.001
Abnormal heart rhythm, *n (%)*	431 (5.8%)	204 (4.7%)	227 (7.6%)	<.001
Stroke, *n (%)*	217 (2.9%)	74 (1.7%)	143 (4.8%)	<.001
Number of CVDs, *n (%)*				<.001
None	6103 (82.7%)	3868 (88.5%)	2235 (74.4%)	
One	945 (12.8%)	392 (9.0%)	553 (18.4%)	
Two or more	329 (4.5%)	112 (2.6%)	217 (7.2%)	
Missing-data pattern				
Completer, *n (%)*	3924 (53.2%)	2553 (58.4%)	1371 (45.6%)	<.001
Non-completer, *n (%)*	3453 (46.8%)	1819 (41.6%)	1634 (54.4%)	
Depression				
CES-D, *mean (SD)*	1.5 (1.9)	1.4 (2)	1.5 (1.9)	.005
Outcome				
Immediate recall, *mean (SD)*	5.70 (1.63)	6.06 (1.55)	5.18 (1.61)	<.001
Delayed recall, *mean (SD)*	4.28 (1.99)	4.74 (1.86)	3.61 (1.98)	<.001
Total score episodic memory, *mean (SD)*	10.0 (3.3)	10.8 (3.1)	8.8 (3.3)	<.001

*Note: SD* Standard Deviation, *CES-D* Center for Epidemiologic Studies Depression Scale, *non CVDs* non-cardiovascular diseases, *CVDs* Cardiovascular diseases. CES-D scores ranged from 0 to 8; immediate and delayed recall ranged from 0 to 10; total score for episodic memory ranged from 0 to 20; low education level included people with no qualifications

Compared with the younger cohort, older participants presented higher rates of low education level (*p* < 0.001), and higher proportions of two or more non-CVDs and CVDs (*p* < 0.001). The prevalence of number of CVRFs was also higher in the older cohort (*p* < 0.0001). Mean score of episodic memory at baseline was lower than middle-aged people (*p* < 0.001). Number of depressive symptoms was also higher (*p* < 0.01). Some 22.5% of older participants died by the end of the study, and 45.6% completed all the assessments.

### Trajectories of verbal memory score

Table 2 shows parameters from unadjusted and adjusted models in both samples. In the unadjusted models, there was a significant dose-response cross-sectional association between number of CVRFs and baseline memory function in both middle-aged and older adults, with higher number associated with lower memory scores (*p* < 0.0001). Longitudinally, there was a significant improvement in memory scores over time in middle-aged participants (*p* < 0.05), whereas a decline was observed in older participants (*p* < 0.0001).

**Table 2:** Parameter estimates, Standard Errors and *p* values from adjusted linear mixed regression models for episodic memory scores

		Unadjusted models						Adjusted models					
Parameter	Categories	Middle-aged			Older adults			Middle-aged			Older people		
		Estimation	SE	*p*	Estimation	SE	*p*	Estimation	SE	*p*	Estimation	SE	*p*
Intercept								**8.384**	**0.220**	**<.0001**	**6.60**	**0.311**	**<.0001**
Time (per year)		**0.010**	**0.005**	**.039**	**−0.115**	**0.007**	**<.0001**	**0.040**	**0.011**	**.001**	**−0.096**	**0.019**	**<.0001**
CVRFs	None	Ref.			Ref.			Ref.			Ref.		
	One	**−0.470**	**0.105**	**<.0001**	**−0.376**	**0.161**	**.020**	− 0.056	0.106	0.599	0.139	0.157	0.378
	Two	**−1.101**	**0.115**	**<.0001**	**−0.792**	**0.166**	**<.0001**	**− 0.245**	**0.118**	**0.038**	0.111	0.164	0.498
	Three or more	**−1.463**	**0.131**	**<.0001**	**−1.414**	**0.174**	**<.0001**	−0.241	0.137	0.080	−0.194	0.175	0.268
Age (centred)		**−0.132**	**0.009**	**<.0001**	**−0.197**	**0.012**	**<.0001**	**−0.110**	**0.008**	**<.0001**	**−0.153**	**0.011**	**<.0001**
CES-D		**−0.248**	**0.019**	**<.0001**	**−0.246**	**0.027**	**<.0001**	**−0.149**	**0.018**	**<.0001**	**−0.119**	**0.025**	**<.0001**
Missing-data pattern	Non-completers	Ref.			Ref.			Ref.			Ref.		
	Completers	**0.961**	**0.077**	**<.0001**	**1.602**	**0.097**	**<.0001**	**0.550**	**0.069**	**<.0001**	**1.090**	**0.091**	**<.0001**
Wealth	1-quintile (lowest)	Ref.			Ref.			Ref.			Ref.		
	2-quintile	**0.641**	**0.076**	**<.0001**	**0.849**	**0.156**	**<.0001**	0.221	0.121	.068	**0.515**	**0.143**	**<.0001**
	3-quintile	**1.265**	**0.128**	**<.0001**	**1.340**	**0.155**	**<.0001**	**0.532**	**0.123**	**<.0001**	**0.780**	**0.146**	**<.0001**
	4-quintile	**1.761**	**0.126**	**<.0001**	**1.728**	**0.153**	**<.0001**	**0.746**	**0.124**	**<.0001**	**0.875**	**0.150**	**<.0001**
	5-quintile (highest)	**2.105**	**0.124**	**<.0001**	**2.476**	**0.155**	**<.0001**	**0.973**	**0.128**	**<.0001**	**1.371**	**0.159**	**<.0001**
Gender	Males	Ref.			Ref.			Ref.			Ref.		
	Female	**0.668**	**0.076**	**<.0001**	**0.612**	**0.101**	**<.0001**	**0.863**	**0.069**	**<.0001**	**0.855**	**0.094**	**<.0001**
Education level	Low	Ref.			Ref.			Ref.			Ref.		
	Medium	**1.470**	**0.090**	**<.0001**	**1.375**	**0.113**	**<.0001**	**1.034**	**0.087**	**<.0001**	**0.964**	**0.106**	**<.0001**
	High	**2.267**	**0.087**	**<.0001**	**2.055**	**0.122**	**<.0001**	**1.711**	**0.090**	**<.0001**	**1.443**	**0.122**	**<.0001**
Marital status	Never married	Ref.			Ref.			Ref.			Ref.		
	Married/remarried	**0.555**	**0.167**	**.001**	**0.885**	**0.245**	**<.0001**	0.255	0.146	.082	0.353	0.214	.099
	Legally separated or divorced	0.262	0.190	0.167	**0.733**	**0.297**	**0.014**	0.295	0.166	.075	0.434	0.258	.092
	Widowed	0.013	0.229	0.954	**0.524**	**0.259**	**0.043**	0.202	0.202	.316	**0.560**	**0.226**	**.013**
Non-CVDs	None	Ref.			Ref.			Ref.			Ref.		
	One	**−0.250**	**0.085**	**.003**	0.017	0.109	0.875	0.050	0.075	.506	0.156	0.096	.106
	Two or more	**−0.546**	**0.135**	**<.0001**	−0.072	0.155	0.641	0.122	0.123	.322	**0.348**	**0.139**	**.013**
CVDs	None	Ref.			Ref.			Ref.			Ref.		
	One	**−0.668**	**0.133**	**<.0001**	**−0.363**	**0.131**	**<.001**	−0.157	0.118	.181	−0.017	0.115	.883
	Two or more	**−1.289**	**0.241**	**<.0001**	**−0.753**	**0.198**	**<.0001**	−0.154	0.216	.478	−0.068	0.175	.695
CVRFs*time	None*time							Ref.			Ref.		
	One*time (per yr)							**−0.035**	**0.014**	**.013**	−0.035	0.022	.122
	Two*time (per yr)							**−0.45**	**0.015**	**.003**	−0.030	0.023	.197
	Three or more*time (per yr)							**−0.061**	**0.018**	**.001**	−0.027	0.025	.286
**Random variance**													
Intercept								**3.438**	**0.186**	**<.0001**	**4.101**	**0.263**	**<.0001**
Linear slope								**0.062**	**0.010**	**<.001**	**0.122**	**0.016**	**<.0001**
Residual								**4.968**	**0.060**	**<.001**	**5.313**	**0.082**	**<.0001**

*Note:* In bold, significant effect

*SE* Standard error, *CVRFs* Cardiovascular risk factors score, *CES-D* Centre for Epidemiologic Studies Depression Scale, *CVDs* Cardiovascular diseases, *non-CVDs* non cardiovascular diseases

CES-D scores ranged from 0 to 8; Episodic memory scores ranged from 0 to 20; low education level included people with no qualifications

After adjusting for covariates, in middle-aged participants, the cross-sectional association between CVRFs at baseline and memory score was no longer significant (*p* = 0.079). Participants who did not miss a study wave had better memory scores than those who missed at least one study wave (*p* < 0.001). Compared with the lowest quintile of wealth, higher levels of wealth were associated with better memory. Females, compared with males, and persons with higher levels of education had significantly better levels of memory (*p* < 0.001).

Regarding longitudinal changes in middle-aged persons, the interaction CVRFs*wave was significant (*p* = 0.004) indicating that different categories of CVRFs presented distinct patterns of decline over time.

For people aged 65–79 years, verbal episodic memory was still affected by time after adjustment, with a decline of 0.10 points every 2 years (*p* < 0.001), whereas the CVRFs were not associated with memory neither cross-sectionally not longitudinally; the interaction term between CVRFs and time were not statistically significant (overall significance *p* < 0.478).

The exclusion of CVDs from the adjusted models yielded similar results, as can be seen in Additional file 1: Table S1.

**Fig. 1:**
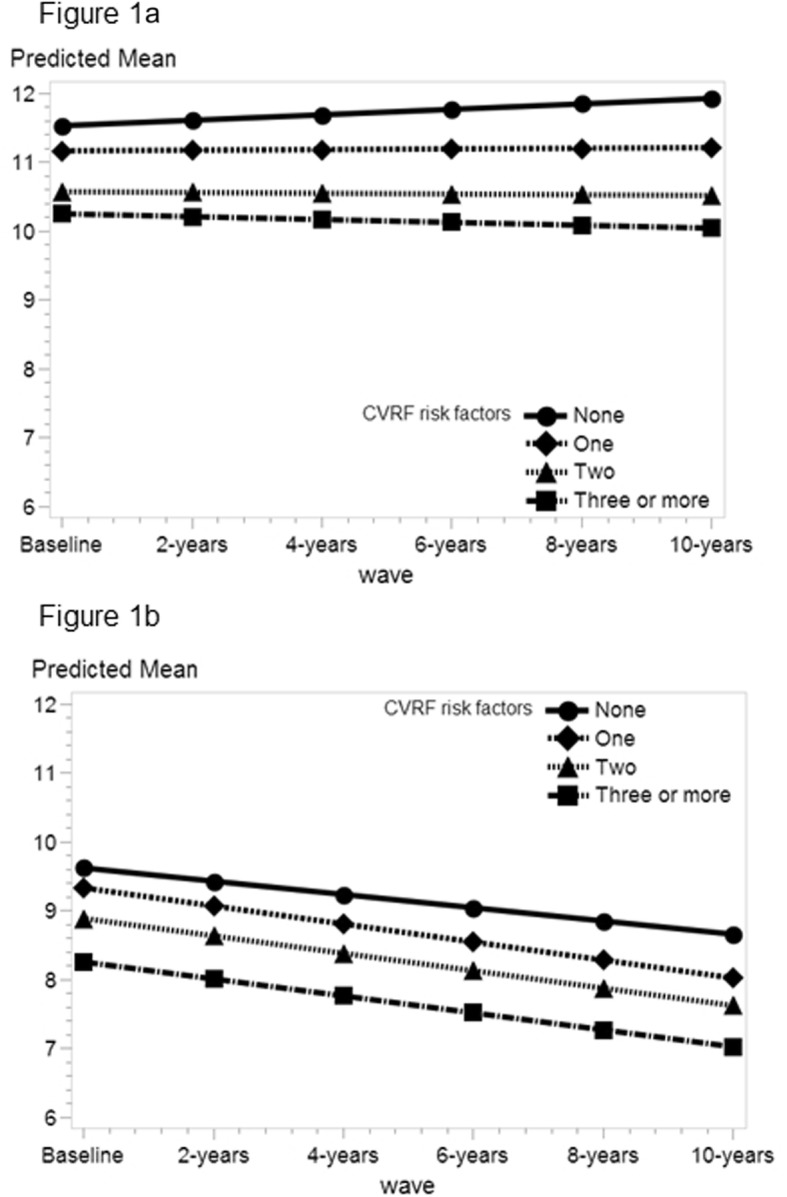
Trajectories of predicted mean for verbal episodic memory in middle-aged and older participants *Note:* Predicted means were calculated from adjusted linear mixed models (see Table 2) while covariates were held constant. CVRFs = cardiovascular risk factors. **a** Middle aged participants, 50–64 years old (*n* = 4372). **b** Older participants, 65–79 years old (*n* = 3005)

Figure 1 shows the trajectories of predicted means of verbal episodic memory over time for each number of CVRFs, stratified by age cohorts and adjusted for covariates. For middle-aged participants (Fig. 1a), those without CVRFs showed a significant improvement in memory scores over time, with a positive slope (*b* = 0.040, SE = 0.012, *p* < 0.01). By contrast, this improvement was not observed in those with *one* (*b* = 0.005, SE = 0.008, *p* = 0.55), *two* (*b* = − 0.005, SE = 0.01, *p* = 0.581) or *three or more* CVRFs (*b* = − 0.021, SE = 0.014, *p* = 0.125). Differences between the slopes of *one*, *two* and *three or more*, compared with *none* CVRFs, were all significant (*p* < 0.05 after Bonferroni correction). There were no significant differences when comparing slopes across *one*, *two* and *three or more* CVRFs.

Figure 1b shows adjusted trajectories of verbal episodic memory for older adults (i.e., aged 65 to 79). Again, slopes for each CVRF were estimated using marginal effects from the adjusted model in Table 2. Older adults with *none* CVRFs presented a significant decline over time (*b* = − 0.096, SE = 0.019, *p* < 0.001). A significant decline was also observed in those with *one* (*b* = − 0.131, SE = 0.012, *p* < 0.001), *two* (*b* = − 0.127, SE = 0.014, *p* < 0.001) and *three or more* CVRFs (*b* = − 0.123, SE = 0.016, *p* < 0.001). However, comparisons between these slopes yielded non-significant differences.

## Discussion

Overall, our findings suggest a greater cardiovascular risk burden on contemporaneous memory decline in midlife, but not in late-life, supporting the hypothesis that the deleterious effect of cumulative CVRFs is age-dependent [10]. They also suggest that the effect of CVRFs follows a dose-response association with verbal episodic memory decline which is independent of other potential confounders.

As has been previously suggested, the presence of CVRFs in midlife is related to increased risk for late-onset cognitive impairment and dementia [27, 28]. Anstey et al. [17] speculated that, if these risk factors for late-life dementia occur in midlife, some effects on cognitive functioning, despite small, might be detectable in middle age. These authors found that greater CVRF burden, measured with a composite score of several risk factors, was associated with faster decline in reaction time. Similarly, other previous longitudinal population-based studies found worse cognitive performance associated with CVRFs in midlife [4, 18, 29]. In our study, middle-aged participants with no CVRFs show an improvement in their memory performance over 10 years. This lack of cognitive deterioration over time, or even some improvement due to practice effect, has been documented in previous research based on general populations, especially among younger and middle-aged adults [17, 30]. However, our results show that participants aged 50 to 64 years who had one, two or three or more CVRFs did not experience this improvement and did significantly worse compared with those free of CVRFs. Albeit subtle, the observed effect of CVRFs on verbal episodic memory is potentially important and meaningful, since one would expect memory not to deteriorate to an observable extent in middle-aged adults [31, 32].

Our findings also indicate that prospective association between cumulative CVRFs and memory in participants aged 50–64 years is dose-response with the memory score getting lower for every additional CVRF. The existing evidence concurs with that and suggests that composites of CVRFs have been shown to have a dose-dependent effect on the risk for dementia [33]. Since these risk factors frequently co-exist, intervention focusing on the combined effect of multiple CVRFs is preferable rather than focusing only on individual risk factors [3].

As for older adults (i.e., aged 65 to 79), we found that there were a significant decline of memory over time, but this was not associated with the number of baseline CVRFs. Previous literature has yielded mixed results when analysing the effect of summary risk scores of CVRFs in later life on risk of dementia, with some population-based studies reporting a positive effect [34, 35], and others failing to report significant effects of CVRFs in late-life on subsequent deterioration of cognitive function [5]. It has been also suggested that CVRFs no longer act as risk factors for dementia [10]. For example, obesity during midlife has been demonstrated to be a risk factor for late-life dementia, whereas for older ages, being underweighted is related to increased risk for cognitive impairment [12]. This U-shaped relationship has been similarly found for hypertension [36]. Higher levels of blood pressure at midlife which decrease over time has been shown to be associated with increased white matter lesions [37]. In a previous study conducted on the ELSA survey [38], we similarly found that the number of CVDs was only significantly related to subsequent latent trajectories of verbal episodic memory in midlife but not in late-life. A recent investigation with the ELSA study also reported that the co-occurrence of diabetes type II and elevated depressive symptoms was associated with accelerated cognitive decline, especially among those aged 50–64 years [39]. Therefore, it is possible that cognitive deterioration in older adults is being affected by different pathways or risk factors. Overall, middle-aged participants present with better levels of health at baseline than older participants. The number of non-CVDs and CVDs were significantly higher in the older cohort, and this might have greatly impact on their memory function. For example, musculoskeletal disease, lung disease or arthritis have been previously linked to cognitive decline [40]. Despite the fact that we controlled for the presence of multimorbidity (including both CVDs and non-CVDs), it is possible that the effect of chronic conditions on participants’ memory function might be more salient on the older cohort than in midlife, thus attenuating the detrimental effect of CVRFs in older participants. There is also the hypothesis that the duration of the exposure to CVRFs might impact on the rate of cognitive decline. Some previous research has supported this when studying diabetes in middle-aged adults [41, 42], hypertension [43], or smoking [44]. Future studies to investigate how age of onset of these CVRFs is impacting on cognitive functioning are warranted.

Survival bias might also partially explain this lack of differences. Presence of CVRFs in younger ages or midlife are related to higher risk for death [45], and thus older participants in our study might be healthier than expected. Cohort effects can also explain this lack of significance. Older and younger cohorts might be exposed to different risk factors or historical events during their lifespan [46].

Some limitations should be considered when interpreting our findings. First, some CVRFs at baseline were self-reported and they could have been affected by recall bias or low accuracy, especially for the older cohort [47]. Second, the age of onset for CVRFs was not considered in this study despite the fact that the duration of these conditions might play an important role on subsequent memory decline. Third, we focused on verbal episodic memory. CVRFs might affect differently the trajectories of other cognitive domains (e.g., attention, verbal fluency). Moreover, one could not discard floor effect, especially in the older age group. Fourth, despite the fact that we excluded those individuals who self-reported being diagnosed with dementia or other brain disorders, it is possible that some others might have presented with milder forms of neurodegenerative disorders, such as mild cognitive impairment (MCI). This would be particularly important if the presence of MCI is contributing to lower baseline cognitive scores and muting the test-retest effect at follow-up, especially in the older group. Finally, non-response might have also introduced some bias. However, we controlled for the confounding effect of completion.

## Conclusions

Our findings support the deleterious effect of aggregate CVRFs in midlife (50–64 years) on subsequent decline over a period of 10 years, whereas this association was not found when CVRFs were measured in older adults (over 65 years). Despite being subtle, the decline observer among middle-aged adults with a high cardiovascular burden was significantly different from that observed for people with no CVRFs, where an improvement was observed. These differences in cognitive decline might increase as people aged, leading to greater risk for future dementia, such as AD. Interventions over these modifiable conditions at midlife could help stop this cognitive deterioration.

## Additional file

**Additional file 1: Table S1.** Parameter estimates, Standard Errors and *p* values from adjusted linear mixed regression models for episodic memory scores (excluding cardiovascular diseases).

## Data Availability

The ELSA dataset is available in the UK Data service repository, https://discover.ukdataservice.ac.uk/catalogue/?sn=5050&type=Data%20catalogue

## Abbreviations

AD: Alzheimer’s Disease
BMI: Body mass index
CES-D: Centre for Epidemiologic Studies Depression Scale
CVD: Cardiovascular disease
CVRFs: Cardiovascular risk factors
ELSA: English Longitudinal Study of Ageing
MCI: Mild Cognitive Impairment
SD: Standard deviation
