# Supplementary material for: Cardiovascular risk factors and memory decline in middle-aged and older adults: the English Longitudinal Study of Ageing
Source: BMC Geriatr. 2019 Dec 2;19:337. doi: 10.1186/s12877-019-1350-5 (PMC6889660; doi:10.1186/s12877-019-1350-5)
Supplement: Supplementary file 1 — Additional file 1: Table S1. Parameter estimates, Standard Errors and p values from adjusted linear mixed regression models for episodic memory scores (excluding cardiovascular diseases). [file 12877_2019_1350_MOESM1_ESM.docx]

Supplemental Table 1 Parameter estimates, Standard Errors and *p* values from adjusted linear mixed regression models for episodic memory scores (excluding cardiovascular diseases)

|  |  | **Adjusted models** | | | | | |
| --- | --- | --- | --- | --- | --- | --- | --- |
| **Parameter** | **Categories** | **Middle-aged** | | | **Older people** | | |
|  |  | Estimation | SE | *p* | Estimation | SE | *p* |
| Intercept |  | **8.678** | **0.196** | **<0,0001** | **6.357** | **0.271** | **<0,0001** |
| Time (per year) | | **0.040** | **0.012** | **0.001** | **-0.096** | **0.019** | **<0,0001** |
| CVRFs | None | ref |  |  | ref |  |  |
|  | One | -0.059 | 0.107 | 0.578 | 0.139 | 0.158 | 0.379 |
|  | Two | **-0.255** | **0.118** | **0.030** | 0.109 | 0.164 | 0.509 |
|  | Three or more | -0.261 | 0.137 | 0.056 | -0.199 | 0.176 | 0.256 |
| Age (centred) |  | **-0.112** | **0.008** | **<0,0001** | **-0.153** | **0.011** | **<0,0001** |
| CES-D |  | **-0.151** | **0.018** | **<0,0001** | **-0.120** | **0.025** | **<0,0001** |
| Missingness | Non-completers | ref |  |  | ref |  |  |
|  | Completers | **0.552** | **0.069** | **<0,0001** | **1.092** | **0.091** | **<0,0001** |
| Wealth | 1-quintile (lowest) | ref |  |  | ref |  |  |
|  | 2-quintile | 0.226 | 0.121 | 0.062 | **0.519** | **0.143** | **0.0003** |
|  | 3-quintile | **0.536** | **0.123** | **<0,0001** | **0.782** | **0.145** | **<0,0001** |
|  | 4-quintile | **0.752** | **0.124** | **<0,0001** | **0.879** | **0.150** | **<0,0001** |
|  | 5-quintile (highest) | **0.983** | **0.127** | **<0,0001** | **1.374** | **0.159** | **<0,0001** |
| Gender | Males | ref |  |  | ref |  |  |
|  | Female | **0.872** | **0.069** | **<0,0001** | **0.859** | **0.094** | **<0,0001** |
| Education level | Low | ref |  |  | ref |  |  |
|  | Medium | **1.034** | **0.087** | **<0,0001** | **0.963** | **0.106** | **<0,0001** |
|  | High | **1.708** | **0.090** | **<0,0001** | **1.441** | **0.122** | **<0,0001** |
| Marital status | Never married | ref |  |  | ref |  |  |
|  | Married/remarried | 0.256 | 0.146 | 0.081 | 0.351 | 0.213 | 0.101 |
|  | Legally separated or divorced | 0.294 | 0.166 | 0.076 | 0.433 | 0.258 | 0.093 |
|  | Widowed | 0.206 | 0.202 | 0.307 | **0.558** | **0.226** | **0.013** |
| Non-CVD | None | ref |  |  | ref |  |  |
|  | One | 0.049 | 0.075 | 0.512 | 0.154 | 0.096 | 0.109 |
|  | Two or more | 0.118 | 0.123 | 0.335 | **0.344** | **0.139** | **0.013** |
| CVRFs*time | None*time | ref |  |  |  |  |  |
|  | One*time (per yr) | **-0.035** | **0.014** | **0.013** | -0.035 | 0.022 | 0.123 |
|  | Two*time (per yr) | **-0.045** | **0.015** | **0.003** | -0.030 | 0.023 | 0.197 |
|  | Three or more*time (per yr) | **-0.060** | **0.018** | **0.001** | -0.027 | 0.025 | 0.286 |
| **Random variance** | |  |  |  |  |  |  |
| Intercept |  | **3.436** | **0.186** | **<.0001** | **4.098** | **0.262** | **<.0001** |
| linear slope |  | **0.062** | **0.010** | **<.0001** | **0.122** | **0.016** | **<.0001** |
| Residual |  | **4.968** | **0.060** | **<.0001** | **5.313** | **0.082** | **<.0001** |

*Note:* In bold, significant effect.

SE=Standard error; CVRFs= Cardiovascular risk factors score; CES-D= Centre for Epidemiologic Studies Depression Scale; non-CVD= non cardiovascular diseases.

CES-D scores ranged from 0 to 8; Episodic memory scores ranged from 0 to 20; low education level included people with no qualifications.
